# The intrapleural volume threshold for ultrasound detection of pneumothoraxes

**DOI:** 10.1186/1757-7241-21-S1-S4

**Published:** 2013-05-28

**Authors:** NP Oveland, E Soreide, F Johannessen, K Wemmelund, R Aagaard, HM Lossius, E Sloth

**Affiliations:** 1 Department of Research and Development, Norwegian Air Ambulance Foundation, Droebak, Norway; 2 Department of Anaesthesiology and Intensive Care, Stavanger University Hospital, Stavanger, Norway; 3 Department of Surgical Sciences, University of Bergen, Bergen, Norway; 4 Department of Radiology, Stavanger University Hospital, Stavanger, Norway; 5 Faculty of Health Sciences, Institute of Clinical Medicine, Aarhus University, Aarhus, Denmark; 6 Department of Anaesthesiology and Intensive Care, Aarhus University Hospital, Aarhus, Denmark

## Objectives

Small pneumothoraxes (PTXs) may not impart an immediate threat to trauma patients after chest injuries. However, if these patients require positive pressure ventilation even a small amount of pleural air may be relevant. Point-of-care lung ultrasonography (US) is a reliable tool in the diagnosis of PTX, but the performance characteristics regarding detection of miniscule PTXs needs to be defined. We aimed at finding the volume threshold of intrapleural air where PTXs confidently can be diagnosed.

## Methods

Air was insufflated into a unilateral pleural catheter in seven incremental steps (10, 25, 50, 100, 200, 350 and 500 mL) in twenty intubated porcine models, followed by a diagnostic evaluation with US and a supine anteroposterior chest radiograph (CXR). The sonographers continued the US scanning until the PTXs could be ruled in, based on identification of the US sign “lung point”. The corresponding threshold volume was noted. A senior radiologist interpreted the CXRs images.

## Results

The mean threshold volume to detect miniscule PTXs using US was 17.8 mL ± 12.8 mL, range 10 mL to 50 mL. Sixty-five % of the PTXs were diagnosed at 10 mL, 25% at 25 mL and the last 10 % at 50 mL of intrapleural air. The radiologist correctly diagnosed 266 lungs (71.1%), had 93 false negative and 15 false positive interpretations. The sensitivity was 31.1% and the specificity 93.8%; the 95% confidence intervals were (22.6, 38.7) and (89.7, 96.4), respectively.

## Conclusion

Miniscule PTXs could be diagnosed with a high level of accuracy using lung US; thus recommended performed by clinicians treating chest trauma patients when PTX is among the differential diagnoses.

## Abbreviations

PTX: pneumothorax; US: Ultrasound; CXR: Chest radiography; CT: Computed tomography

